# Congenital ocular toxoplasmosis in consecutive siblings

**DOI:** 10.5935/0004-2749.20220079

**Published:** 2025-02-11

**Authors:** Milena Simões F. Silva, Aparecida Y. Yamamoto, Cristina G. Carvalheiro, Michael E. Grigg, Ana Leonor A. de Medeiros, Marisa M. Mussi-Pinhata, João M. Furtado

**Affiliations:** 1 Division of Ophthalmology, Faculdade de Medicina de Ribeirão Preto, Universidade de São Paulo, Ribeirão Preto, SP, Brazil; 2 Department of Pediatrics, Faculdade de Medicina de Ribeirão Preto, Universidade de São Paulo, Ribeirão Preto, SP, Brazil; 3 Molecular Parasitology Section, Laboratory of Parasitic Diseases, National Institute of Allergy and Infectious Diseases, National Institutes of Health, Bethesda, MD, USA

**Keywords:** Toxoplasmosis; ocular/congenital, Toxoplasmosis; ocular/genetics, Toxoplasmosis; congenital, Toxoplasma gondii, Uveitis, Toxoplasmose ocular/congênita, Toxoplasmose ocular/genética, Toxoplasmose congênita, *Toxoplasma gondii;* Uveite

## Abstract

*Toxoplasma gondii* infection can cause ocular manifestations after acquired and congenital disease. We report two cases of symptomatic congenital toxoplasmosis with ocular involvement in non-twin siblings, with a 2-year interval between pregnancies. Vertical transmission of toxoplasmosis in successive pregnancies, which was once considered impossible, is now found to be plausible even in immunocompetent subjects.

## Abstract (pt)

A infecção pelo *Toxoplasma gondii* pode causar manifestações oculares tanto após a sua forma congênita quanto a sua forma adquirida. Reportamos aqui dois casos de toxoplasmose congênita sintomática com envolvimento ocular em irmãos não gêmeos, com intervalo de 2 anos entre gestações. A transmissão vertical da toxoplasmose em gestações sucessivas, outrora considerada impossível, é um evento plausível mesmo em indivíduos imunocompetentes.

## INTRODUCTION

*Toxoplasma gondii* is a common human protozoan parasite transmitted mainly through the ingestion of undercooked contaminated meat, water, or vegetables containing oocysts, or vertically^(1)^. *T. gondii* infection can cause ocular manifestations after acquired and congenital disease^(1)^. Congenital infection occurs usually during pregnancy of a woman previously unexposed to the infection^(1)^ or rarely after reactivation of chronic toxoplasmosis during pregnancy^(2-4)^. The possibility of congenital infection occurring after reinfection of a previously seropositive pregnant woman with a different, more virulent *Toxoplasma* strain has also been described^(5)^. Herein, we report an unusual case of symptomatic congenital toxoplasmosis with ocular involvement in non-twin siblings.

## CASE REPORT

**Figure 1:**
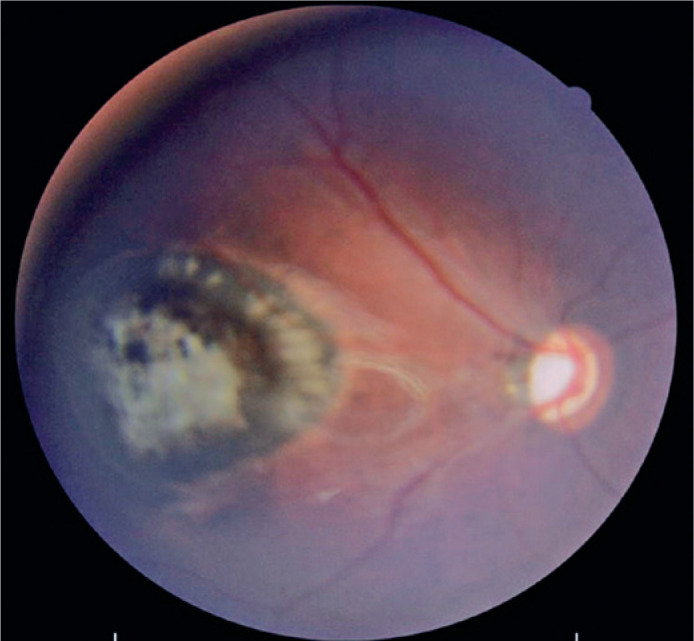
Color fundus photograph of the right eye of the first-born female infant, showing a 3-disc-diameter hyperpigmented retinochoroidal scar in the macula, with central atrophy.

A female infant was born at 36 weeks of gestation, after the identification of maternal toxoplasmosis seroconversion with positive specific IgG and IgM and an avidity index of 7% between 10 and 33 weeks of gestation. Gestational treatment was not initiated because the mother had no further prenatal visits before delivery. The infant was born with a birth weight of 2,675 g and head circumference of 30 cm (above the third percentile), and laboratory investigation revealed high titers of anti-*Toxoplasma* IgG (>300 UI/mL; positive reference range, >8 UI/mL) and positive IgM (3.25 UI/mL; positive reference range, >0.65 UI/mL) in peripheral blood. Neonatal screening results were negative for other infections or metabolic abnormalities. Computed tomography (CT) of the brain revealed multiple parenchymal calcifications associated with hydrocephalus. Ophthalmological evaluation evidenced a hyperpigmented retinochoroidal scar in the macula of the right eye (OD), compatible with a toxoplasmic retinochoroidal scar (Figure 1), and temporal optic nerve pallor in the left eye (OS). Twenty months later, the mother again gave birth, this time to a male newborn.

**Figure 2:**
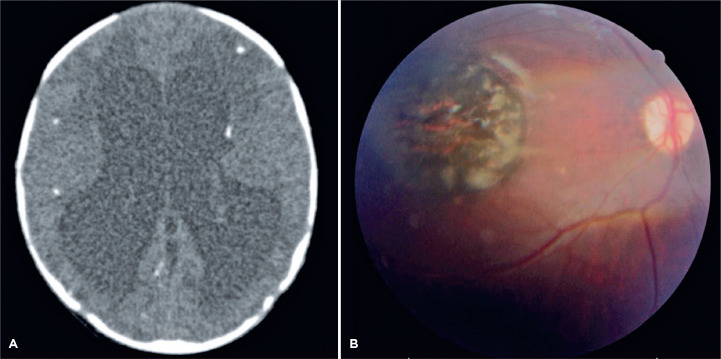
(A) Computerized tomography scan of the brain showing ventricular dilation and cerebral calcifications in the second-born male infant. (B) Color fundus photograph of the right eye of the same patient, showing a round hyperpigmented retinochoroidal lesion in the macular area, compatible with the retinal scar of ocular toxoplasmosis.

Maternal anti-*Toxoplasma* IgG titers were high throughout the second gestation, while the IgM titers ranged from negative to weakly positive in the second trimester, when the IgG avidity was high (83.3%). Again, no gestational treatment was initiated. No abnormalities were detected in the infant right after birth, and no further investigation was performed. However, at 4 months of age, he was admitted to the emergency department with vomiting, drowsiness, and a bulging fontanelle. A brain CT scan evidenced ventricular dilation and cerebral calcifications (Figure 2A). Serological evaluation of the infant revealed positive anti-*Toxoplasma* IgG (>300 UI/ml) and anti-*Toxoplasma* IgM (1.25 UI/ml), and *Toxoplasma* DNA was detected in a cerebrospinal fluid sample analyzed using polymerase chain reaction (PCR). Other congenital infections were ruled out. Ophthalmological examination of the infant revealed a macular pigmented lesion with 2-disc diameters, compatible with the toxoplasmic retinochoroidal scar in the OD (Figure 2B). Both infants were treated with sulfadiazine, pyrimethamine, and folinic acid for 12 months. The diagnosis of congenital toxoplasmosis was confirmed by the positive IgG titers persisting after 1 year of age in both infants. Other maternal infections were ruled out in both pregnancies, including human immunodeficiency virus (HIV). Maternal ocular examination revealed no retinal lesions attributable to toxoplasmosis.

## DISCUSSION

Congenital transmission of toxoplasmosis from previously infected mothers, though rare, is plausible. Silveira et al. described a 38-year-old woman with a previous diagnosis of ocular toxoplasmosis and anti-*T. gondii* IgG+ and IgM- serology who gave birth to a male newborn with anti-*T. gondii* IgG+ and IgM+ and a macular scar compatible with ocular toxoplasmosis^(3)^. Similarly, Andrade et al. described a woman who had reactivation of ocular toxoplasmosis during pregnancy and gave birth to a boy, who presented anti-*T. gondii* IgG+ and IgM+, and multiple and bilateral peripheral retinal active lesions^(2)^.

In the present report, we confirmed the diagnosis of congenital toxoplasmosis in two subsequent siblings born to a mother without typical toxoplasmosis retinal lesions. This case differs from most previous cases because the mother gave birth to two symptomatic infants, both with ocular and neurological involvements. A potential explanation for the symptomatic congenital infection in the second-born infant would be delayed parasite clearance, with persistence of circulating parasites during the second pregnancy within a 2-year interval. The mother was HIV negative, but primary immunodeficiencies, which could also lead to recurrent or persistent parasitemia, were not investigated. Other possible explanations are the reactivation of occult cysts induced by transient pregnancy-related immunosuppression or maternal reinfection by a different *T. gondii* strain. Despite the possibility of a false-positive IgM or the persistence of IgM in chronic toxoplasmosis^(6)^, the new positivity of maternal IgM after its negativity supports the hypothesis of reinfection in the second pregnancy. Postnatal acquired ocular toxoplasmosis^(7)^ is unlikely because the association of ocular and neurological abnormalities is characteristic of congenital toxoplasmosis and the second infant was exclusively breastfed at the time of diagnosis, which reduced the risk of infection through ingestion of contaminated food or water.

To investigate the hypothesis of maternal reinfection, strain typing was attempted through multilocus PCR genotyping in the second infant but was unsuccessful. Enzyme-linked immunosorbent assay-based serotyping was then used to assess the reactivity of the mother and infants to peptides derived from the genotypic markers GRA6 and GRA7 (Table 1)^(8)^. The mother and her children possessed an equivalent *T. gondii* serotype, which supports the idea that reinfection did not occur but did not rule out reinfection by a different strain, as different strains can have similar reactivities to a peptide^(9)^. A limitation of the serotyping assay used for the patients described herein is that it evaluated the reactivity to peptides derived from two genotypic markers that are not the sole determinants of parasite virulence^(9)^. The serotype possessed reactivity to type II strain epitopes, which are uncommon in Brazil, where atypical and diverse *T. gondii* genotypes are found^(9)^, and not associated with severe ocular toxoplasmosis in Europe^(10)^.

**Table 1:** *Toxoplasma gondii* serotyping of the mother and first (child 1) and second (child 2) offspring

Peptide^a^	Mother	Child 1	Child 2	Cutoff^d^
6 I/III	1.59	2.07	1.15	2,68
d6 I/III	0.94	1.24	0.98	1,53
6 II	4.42^*^	4.81^*^	3.28^*^	1,47
7 II	5.41^*^	3.28^*^	1.17	1,58
SAG1^b^	6.57^*^	7.28^*^	6.52^*^	0,88
**Toxoplasma serotype** ^c^	**II**	**II**	**II**	-

* = The values refer to the mean optical density index obtained for each sample; positive values are marked with an asterisk.

a = peptides are named as follows: “6” or “7,” peptides derived from the genotypic markers GRA6 or GRA7; “I/III” or “II,” the archetypal parasite strain from which the peptide was derived; and “d,” a truncated diagnostic peptide;

b = SAG1 indicates the presence of anti-Toxoplasma antibodies;

c = infections by type I/III strains produce antibodies that react with either or both 6 I/III and d6 I/III peptides. Infections by the type II strains produce antibodies that react with either or both 6 II and 7 II peptides. Atypical serotypes react with both type I/III and II peptides or do not react with any peptide;

d = calculated from the mean values + 2 standard deviations obtained from 41 healthy pregnant women who were seronegative for toxoplasmosis.

In conclusion, congenital toxoplasmosis in infants from previously seropositive mothers can occur and may cause severe ocular and central nervous system involvements. Although the frequency and mechanisms of the mother-to-fetus transmission of toxoplasmosis in previously seropositive women are unclear, our findings could alert clinicians to reinforce preventive measures such as avoiding exposure to *T. gondii* sources for all pregnant women independently of their immune status, properly treating *Toxoplasma* infection in pregnant women, and postponing gestation in those with a recent infection, although the length of the appropriate waiting time is unclear.
